# EmerSense: A low-cost multiparameter logger to monitor occurrence and duration of emersion events within intertidal zones

**DOI:** 10.1016/j.ohx.2023.e00410

**Published:** 2023-03-01

**Authors:** Rosa Celia Poquita-Du, Ian Peter Morgia Du, Peter A. Todd

**Affiliations:** Experimental Marine Ecology Laboratory, S3 Level 2, Department of Biological Sciences, National University of Singapore, 16 Science Drive 4, Singapore 117558, Singapore

**Keywords:** Arduino microcontroller, Time of flight sensor, Coral emersion, Coastal monitoring, Data acquisition

## Abstract

The intertidal zone is a harsh environment for marine life as conditions are often both extreme and variable. A wide range of sessile organisms are partially or fully emersed (exposed above the water line) during low tide. In the tropics, when corals are emersed, high light and temperature can be detrimental to their survival. To date, there is no commercially-available logger that can detect periods of emersion, information that is useful for marine research and for coastal resource management. Here, we present a low-cost Arduino-based multi-parameter logger called ‘EmerSense’ which can detect instances of emersion while simultaneously recording light and temperature profiles. We describe the different steps involved in fabricating EmerSense, including hardware construction and software design, and discuss the results of our field testing at an intertidal coral reef in Singapore.

## Graphical abstract

**Figure:**
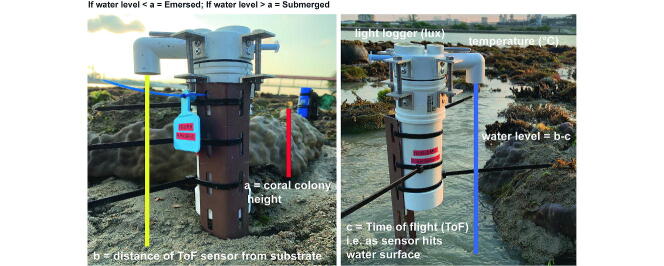

## Hardware in context

Organisms living within the intertidal zone experience highly variable and challenging environmental conditions associated with tidal fluctuations, including changes in light intensity, temperature, and salinity [1], [2]. Sessile organisms can be partially or completely exposed (emersed) during spring low tide, subjecting them to sub-optimal conditions [3], [4]. There is limited information regarding the duration of emersion these organisms experience, as well as the magnitude of the environmental stressors they are exposed to [5], [6]. Being able to measure this information *in situ* facilitates further examination of the limits to which intertidal species can survive periods of emersion.

In Singapore, intertidal corals are common, but poorly studied. Monitoring intertidal emersion duration is not only key to understanding environmental dynamics, it is also critical for the design of restoration projects—particularly those involving coral transplantation to enhance biodiversity on artificial structures such as seawalls [5], [6], [7]. However, there is currently no commercially-available logger that can monitor periods of emersion. Instead, estimates of exposure in the intertidal environment are generally derived from daily tidal height projections [5], [6] which vary in accuracy and generally cover large areas (kilometres) of coastline. They do not provide accurate emersion periods for locations at smaller (meters) scales.

Here, we describe an Arduino-based underwater multiparameter logger that records the instances and duration of emersion while simultaneously logging light and temperature, which are two important factors affecting sessile organisms living between the tides. Many of the existing non-commercial logger designs for other environmental parameters also utilize an Arduino-based microcontroller as this represents one of the more affordable options in the market, with an extensive network of support in terms of modules and libraries. Additionally, Arduino is known to withstand harsh environments, making it ideal for deployment within intertidal areas. Our design includes a low-cost, waterproof housing made from components that can be bought off-the-shelf from hardware stores. Here, we describe the different steps involved in fabricating the multiparameter logger: ‘EmerSense’, including hardware and software design, and discuss the results of our field testing at an intertidal coral reef in Singapore.

## Hardware description

EmerSense is constructed from readily available and low-cost materials. It utilizes an Arduino microcontroller that can be programmed easily using the provided source code (Table 1), with some flexibility to add or adjust parameters to suit specific research objectives. All the electronic components are housed in a Polyvinyl Chloride (PVC) cylindrical container with O-ring fitted caps clamped down with stainless steel bolts and nuts. The logger is powered by four AA batteries and measurements of parameters are saved in a storage device (SD) card. Our design utilized the time-of-flight (ToF) principle to establish the distance of an object through determination of the water level at particular time points using a ToF sensor. Water levels can be derived from calculations of the differences between the fixed distance of the ToF sensor to the substrate (i.e. the height it is installed) and distances recorded by the sensor every time it hits the water surface. Particularly, we used a ToF laser-ranging module (VL53L0X) which provides accurate distances regardless of the reflectance of the target object.

**Table 1:** contains links to the files required to construct EmerSense.

Design file name and type	Open source license	Location of the file	
EmerSense components (.fig)	CC Attribution 4.0 International	https://doi.org/10.5281/zenodo.5158903	
EmerSense source code (.ino)	CC Attribution 4.0 International	https://doi.org/10.5281/zenodo.5158903	

## Design files

### Bill of materials

The components required to build one unit of EmerSense, and the corresponding costs are provided in Table 2 below. The majority of the materials were locally-sourced so the costs presented below were based from current conversion of Singapore dollars (SGD) to United States dollars (USD), i.e. 1 SGD = 0.74 USD [8].

**Table 2:** Individual components required for building one unit of EmerSense.

Material	Description	Number	Cost per unit (USD)	Total Cost (USD)
Donut board	6 × 15 cm	1	0.42	0.42
Strip board	6 × 14 cm	1	0.42	0.42
Resistor	0.25 W 5% (1 K)	1	0.01	0.01
Straight pin headers	female (1 × 40 ways) male (1 × 40 ways)	1	0.16	0.16
Compact battery holder	for 4 × AA batteries	1	0.32	0.32
2510 PCB connector housing	2 ways	2	0.53	1.05
2510 PCB connector housing	3 ways	1	0.02	0.02
2510 PCB connector housing	4 ways	2	0.03	0.06
2600 PCB connector header (R/A)	2 ways	2	0.04	0.09
2600 PCB connector header (R/A)	3 ways	1	0.07	0.07
2600 PCB connector header (R/A)	4 ways	2	0.08	0.16
Terminal pin	for 2510	15	0.03	0.44
Arduino header	female (1 × 8 ways)	4	0.32	1.27
Temperature sensor	waterproof	1	2.12	2.12
NANO compatible (CH340)	with USB cable	1	6.87	6.87
RTC I^2^C module	DS1307	1	0.93	0.93
Water float/level sensor switch	–	1	2.17	2.17
Micro SD card adapter	5 V	1	1.30	1.30
Time of flight (ToF) distance sensor module	VL53L0X V2 laser	1	6.61	6.61
Light intensity sensor module	Digital; BH1750FVI	1	2.35	2.35
Silicone wire kit	22AWG 600 V; tinted, 6-color	1	10.31	10.31
Hook up wire kit	22AWG; solid tinted; 6-color	1	10.31	10.31
PVC end cap	Diameter (D) = 50 mm	2	2.52	5.03
PVC coupling	D = 50 mm	1	1.48	1.48
PVC pipe	D = 50 mm; Length (L) = 30 cm	1	1.33	1.33
PVC pipe	D = 32 mm; L = 30 cm	1	0.89	0.89
PVC pipe	D = 18 mm	1	7.00	7.00
Bolts	stainless steel	4	0.67	2.68
Nuts and washers	stainless steel	4	0.45	1.80
Rivets	1 pack	16	1.07	1.07
Epoxy	two-part	1	2.03	2.03

## Build instructions

Building the EmerSense requires some handling of power tools, basic skills in electronics, and simple programming. The construction process involves three main steps: 1) assembling the electronic components (2) creating the Arduino-based program and, (3) building the waterproof housing.

### Assembling the electronics component

**Fig. 1:**
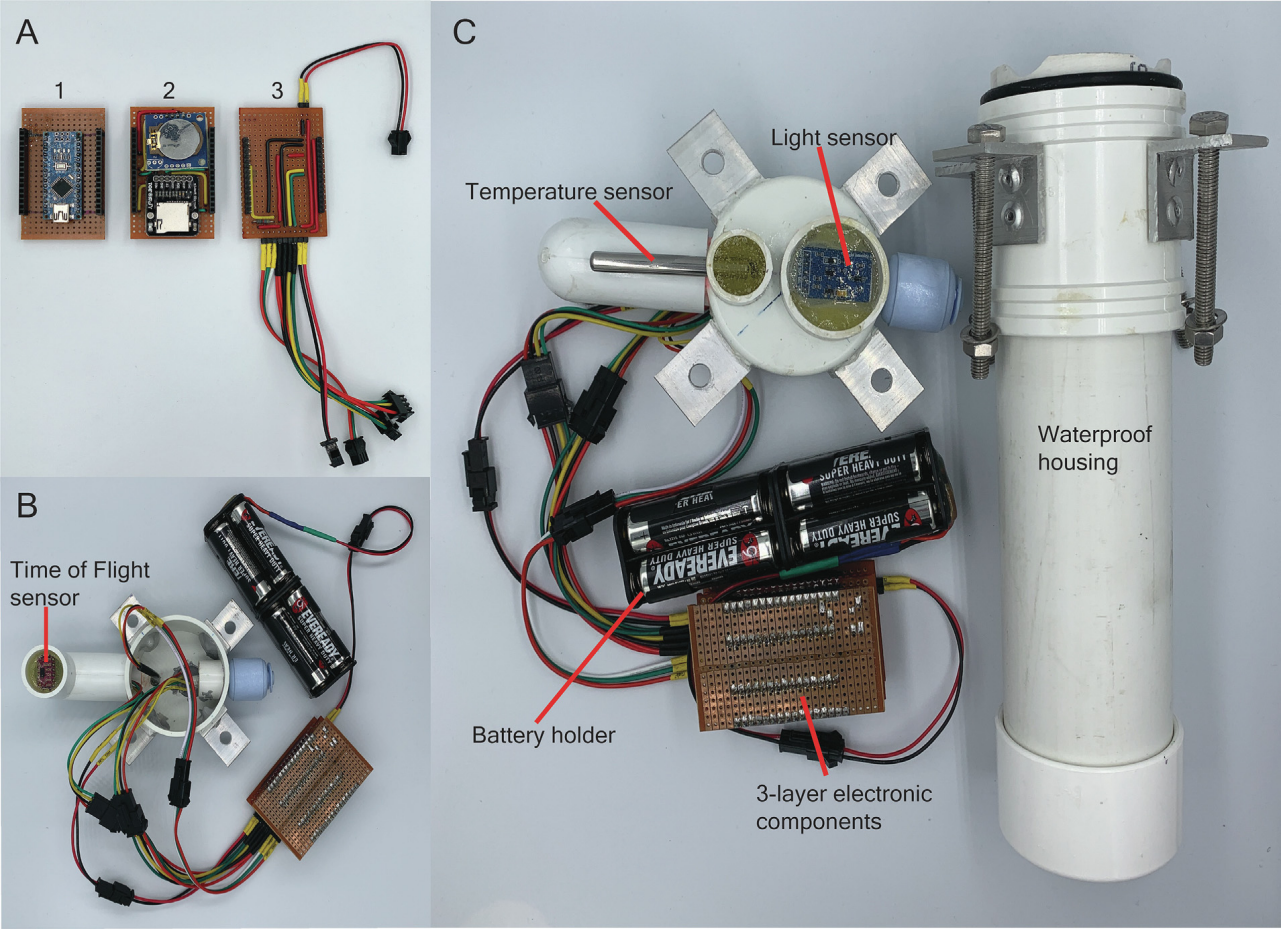
The EmerSense multiparameter logger consists of a 3-layer electronic component (A) and three environmental sensors: Time of Flight, Light and Temperature (B-C). All of the components are wired to a battery holder that is powered by four AA batteries and housed in a PVC-made waterproof housing, pressure-sealed with threaded bolts and nuts.

**Fig. 2:**
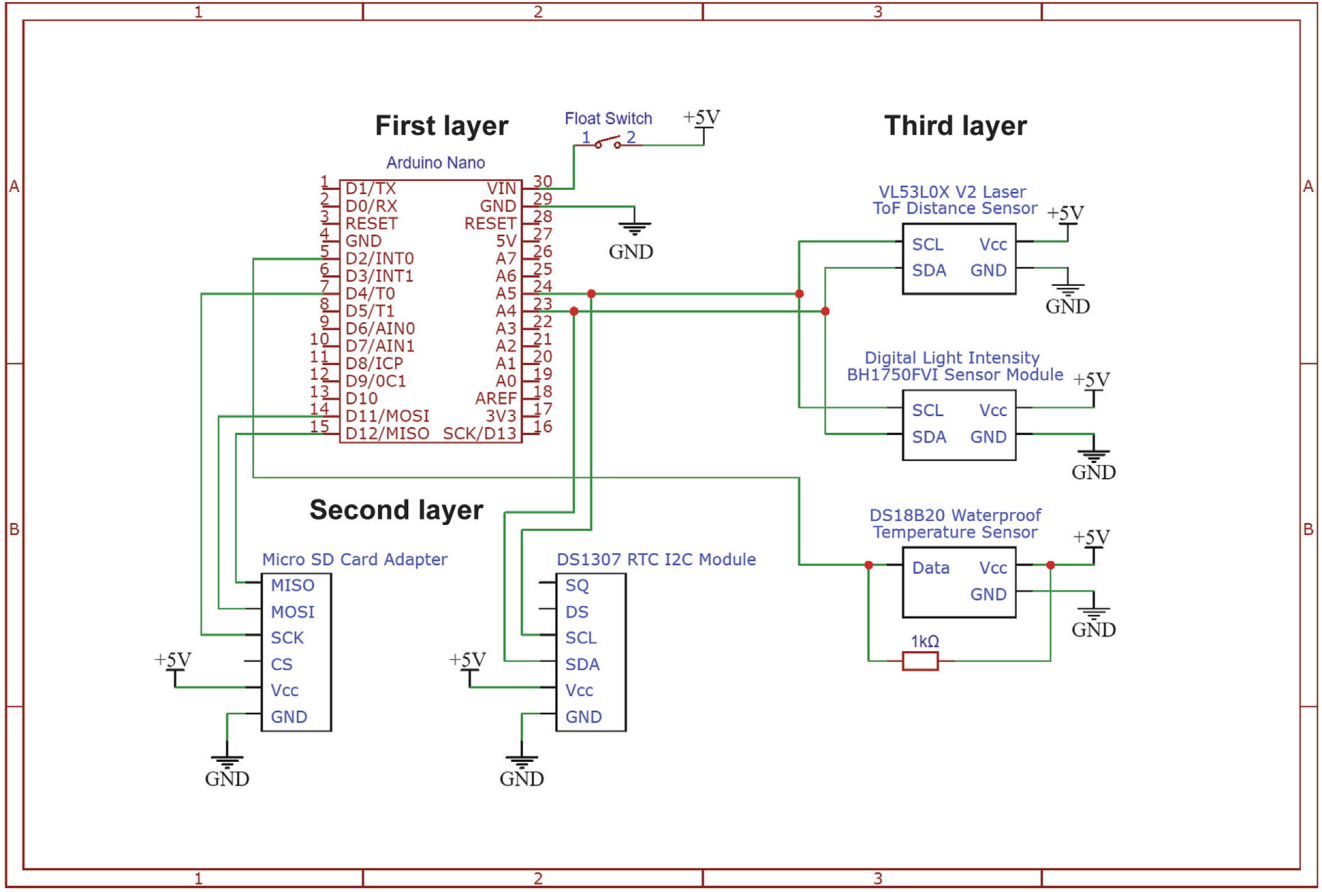
Circuit wiring diagram showing the three different layers of EmerSense’s electronic component.

The EmerSense’s electronic component consists of three layers with the first layer being the microcontroller (Fig. 1A.1, Fig. 2), second is the logger (Fig. 1A.2) and the third is the sensor routing layer (Fig. 1A.3). The microcontroller used in the EmerSense is an Arduino nano compatible board (CH340). A strip board is first cut into a 4 cm × 6.5 cm rectangle with its contact strips orientated perpendicular to the microcontroller. The contacts at centre of the strip board are scraped off with a Dremel tool to create 2 separate channels (left and right). The microcontroller and two straight female headers (1 × 16 dimension) are then soldered onto the strip board. On the top and bottom of the board, the contacts between 2 pin holes are scraped off using a utility cutter and then soldered to two 2-way right-angled 2600 printed circuit board (PCB) connector headers. A modified float on/off switch (by means of an Infrared system), power supply and, two sets of compact 4 × AA battery holder (Fig. 1B) are glued end-to-end and wired in parallel.

The second layer (Fig. 1A.2) contains the components needed to store and accurately log the data collected by EmerSense. This layer comprises a real time clock (RTC) I^2^C Module, a 5V compatible micro storage device (SD) card adapter, and four female headers (1 × 8 dimension) with extra-long legs. A donut board is cut into a 4 cm × 6.5 cm rectangle with two of the headers soldered to each of the long sides. The RTC module’s pins are connected to the board as follows: voltage common collector (VCC) to 5V, ground (GND) to GND, serial data (SDA) to A4 and serial clock (SCL) to A5. The SD Card Adapter’s pins are connected to the board as follows: VCC to 5V, GND to GND, (master out slave in (MOSI) to D11, master in slave out (MISO) to D12 and SCK to D13.

The sensor routing component (i.e. the third layer, Fig. 1A.3) contains the headers that connect the sensors to the microcontroller in the first layer. The third layer comprises two 2-way right-angled 2600 PCB connector headers, one 3-way right-angled 2600 PCB connector header and, a 1 K ohm resistor for the temperature sensor (Fig. 1C). The two 4-way connectors are used to connect to the VL53L0X V2 laser time-of-flight (ToF) distance sensor (Fig. 1B) module and digital light intensity BH1750FVI sensor module (Fig. 1C). These two modules use I^2^C so both the 4-way headers will be wired as follows: VCC to 5v, GND to GND, SDA to A4 and SCL to A5. The same order of the pins for the 4-way connector are used to solder the connections on the 4-way 2510 PCB connector housing. A single 3-way right-angled 2600 PCB connector header is used to connect the DS18B20 waterproof temperature sensor using a 1-wire interface. Finally, the 3-way header (i.e. female connector) is wired as follows: VCC to 5v, GND to GND, Data to D3. The same order is soldered on the 3-way 2510 PCB connector housing for the male pins.

### Creating the Arduino based program

Programming of the EmerSense is performed using the Arduino Integrated Development Environment (IDE). The following libraries are used to support the desired functionalities:

1. SD.h – allows for reading from and writing to SD cards. It supports FAT16 and FAT32 file systems on standard SD cards and SDHC cards.
2. SPI.h – to communicate with serial peripheral interface (SPI) devices with Arduino as the master device
3. BH1750.h - for digital light sensor breakout boards containing the BH1750FVI IC.
4. Wire.h – to communicate with Inter-integrated Circuit (I^2^C) devices
5. DallasTemperature.h - allows the use of temperature sensors and supports multiple sensors, one of which is the DS18B20 waterproof temperature sensor
6. VL53L0X.h - for the Arduino IDE that helps interface with ST's VL53L0X time-of-flight distance sensor. The library makes it simple to configure the sensor and read range data from it via I2C.
7. DS1307RTC.h - functions with any module that uses the DS1307 RTC chip which provides the EmerSense access to real-time clock functionalities.
8. TimeLib.h - to compliment the DS1307RTC library and adds a number of time-specific functions.

In the source code, the variable “delayVal” contains the logging interval in milliseconds and can be edited to suit the project’s needs.

### Building the waterproof housing

The waterproof housing (Fig. 1C) is made of varying lengths of 50 mm diameter size white PVC tubing, end caps, coupler, eight 25 mm right angle aluminium cut to 20 mm lengths and 4 × A2-70 stainless steel bolts and nuts with washers. The construction of the housing can be separated into 2 parts: the sensor cap and the main body.

The main body:

1. Cut 40 mm and 90 mm length of the 50 mm white PVC tubing.
2. Glue both tubes together to the 50 mm white PVC coupler and on the longer end (90 mm) glue a 50 mm white PVC end cap.
3. Drill two 4 mm holes on the PVC coupler, 22 mm and 32 mm from the top of the coupler respectively. Then repeat at the 0°, 90°, 180° and 270°.
4. Take the 20 mm length right angle aluminium and drill a 6 mm hole at the center of one side of the angle aluminium, roughly 10 mm from the side and 13 mm from the angled side.
5. Take the 20 mm length right angle aluminium and drill two 4 mm holes on the other side for the angle aluminium, roughly 10 mm from the side and 8 mm and 18 mm from the angle side.
6. Position the angle aluminium and align the two 4 mm holes to the same holes on the PVC coupler. Once aligned, rivet the two holes.
7. Seal the rivets with epoxy from the inside of the housing. Note that if the seam where the coupler has a gap, it also needs to be filled with epoxy.

Sensor Cap:

1. Prepare the VL53L0X V2 Laser ToF Distance Sensor Module by soldering the straight pin headers to the PCB. Take a length of 22 awg wire and on one end, solder it to the straight pin headers and terminal pins on the other. Attach the terminal pins to the four-way 2510 PCB Connector Housing. Cut off the sides to provide a profile that can fit on top of a 20 mm PVC pipe without any of its edges protruding.
2. Prepare the Digital Light Intensity BH1750FVI Sensor Module by soldering the straight pin headers to the PCB. Take a length of 22 awg wire and solder one end to the straight pin headers and a 4-way header (i.e. female connector) on the other end of the wire. Attach the terminal pins to the 2510 PCB Connector Housing 4 Ways. Cut off approximately 7 mm off the PCB.
3. Take a 15 mm PVC elbow and cut one end short by 10 mm. On the shorter side place the VL53L0X V2 Laser ToF Distance Sensor Module with the sensor facing outwards. Fill in the gaps from behind the sensor with some reusable putty (e.g. Blu Tack) and proceed to fill in and cover the sensor with epoxy resin to seal it. Remove the reusable putty once the epoxy resin has hardened.
4. Drill two 4 mm holes on the 50 mm white PVC end cap at 12 mm and 22 mm from the top of the coupler, respectively. Then repeat at the 0°, 90°, 180° and 270° around the cap.
5. Drill a 21 mm hole on the side of the 50 mm white PVC End Cap at 135° and attach a 30 mm length of the 15 mm white PVC pipe using PVC glue. Connect the PVC elbow as in STEP 3 to the extending PVC pipe using PVC glue.
6. Take a 10 mm length of 15 mm white PVC pipe and drill a 6 mm hole on its side and glue it to the top of the 50 mm white PVC end cap at 135°. Drill a 6 mm hole on the area of the end cap that is enclosed by the 15 mm white PVC pipe. Thread through these holes a DS18B20 temperature sensor with most of the sensor protruding from the side. Take some reusable putty and fill in any gaps from inside the PVC end cap and also the outside area of the hole where the temperature sensor is protruding. Fill in the area enclosed by the 15 mm white PVC pipe with epoxy putty to seal it completely.

## Operation instructions

### Deployment

Prior to deployment, the logging interval is set according to the desired frequency by changing the variable “delayVal” in the code. A MicroSD card is inserted in the SD card module to save the logged data. Newly-charged batteries are placed in the holders to ensure that there will be enough power for the entire duration of monitoring. All connectors for the sensors are connected to the Sensor Routing Layer. Finally, the housing is sealed up by tightening the four bolts. The distance of the ToF sensor to the substrate and the height of the reference coral colony is recorded for later calculations of changes in water level throughout the monitoring period. To start logging, the float switch is detached from its base to trigger the “ON” configuration.

### Recovery

Set the EmerSense to its “OFF” configuration by placing the float switch back in to its base. Dry the housing before undoing the bolts and opening it. When completely dried, uncouple the sensor connectors from the Sensor Routing Layer and remove the MicroSD card to retrieve the recorded data. Open the data file in an Excel worksheet and encode the distance of ToF sensor from the substrate where the EmerSense Unit was deployed and the height of the reference colony for calculation of water level to infer instances of emersion (i.e. when water level is lower than the colony height).

## Validation and characterization

### Testing EmerSense in the field

Multiple deployments of the earlier prototypes of EmerSense, without the electronic components, were performed to test the waterproof housing for any leaks. Three initial designs were tested: (1) friction-seal (2) O-ring seal using a screw cap and (3) combination of O-ring and pressure seal using threaded bolts and nuts. The third prototype did not develop any leaks. We performed an additional (pressure) test on this design to 50 psi in water for 15 min using a self-fabricated pressure chamber. Again, no leaks were found.

**Fig. 3:**
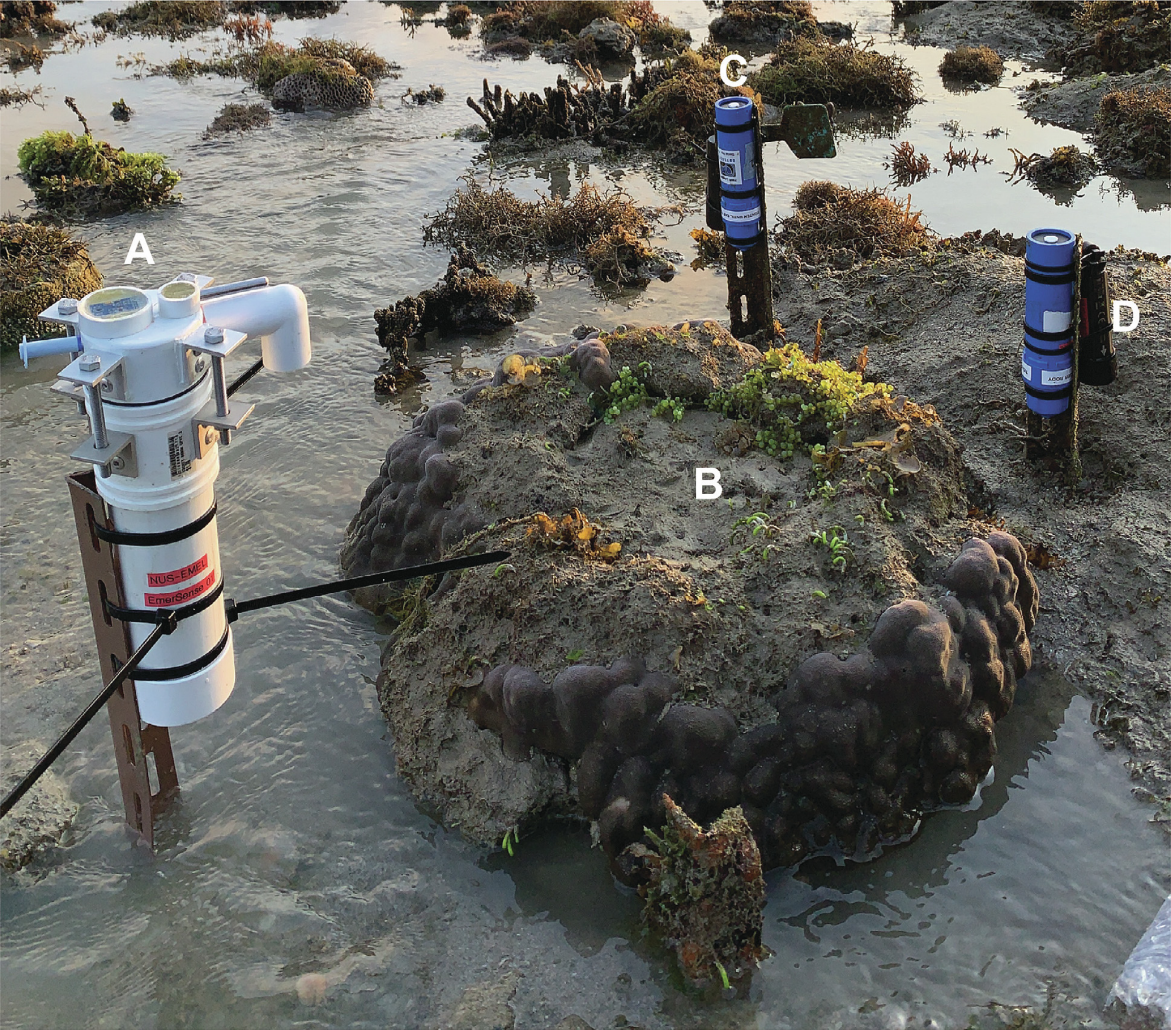
Field setup showing the EmerSense (A) deployed near to a coral colony (B), with two sets of Odyssey light (C) and HOBO temperature (D) loggers.

Here, we present data from two EmerSense units, with all the electronics components inside, that were deployed on the intertidal flat of Pulau Hantu, one of Singapore’s Southern Islands, on 29 April 2021 during the spring low tide period. Each EmerSense was secured onto an angle iron (using cable ties) that had been driven into the substrate adjacent to a massive-form coral colony (Fig. 3). Pairs of the Odyssey photosynthetic irradiance recording system (Dataflow Systems Limited, New Zealand) and HOBO water temperature (U22-001 Pro v2, Onset Computer Corporation, USA) data loggers were also placed near the same colony for continuous monitoring of the surrounding conditions. The Odyssey light sensor measures light levels within the photosynthetically active radiation (PAR) range only. All loggers were left in the field to log every 2 min for 24 h. Readings from two units of HOBO temperature and two units of Odyssey light loggers were each averaged and, compared with the environmental profiles recorded by EmerSense.

**Fig. 4:**
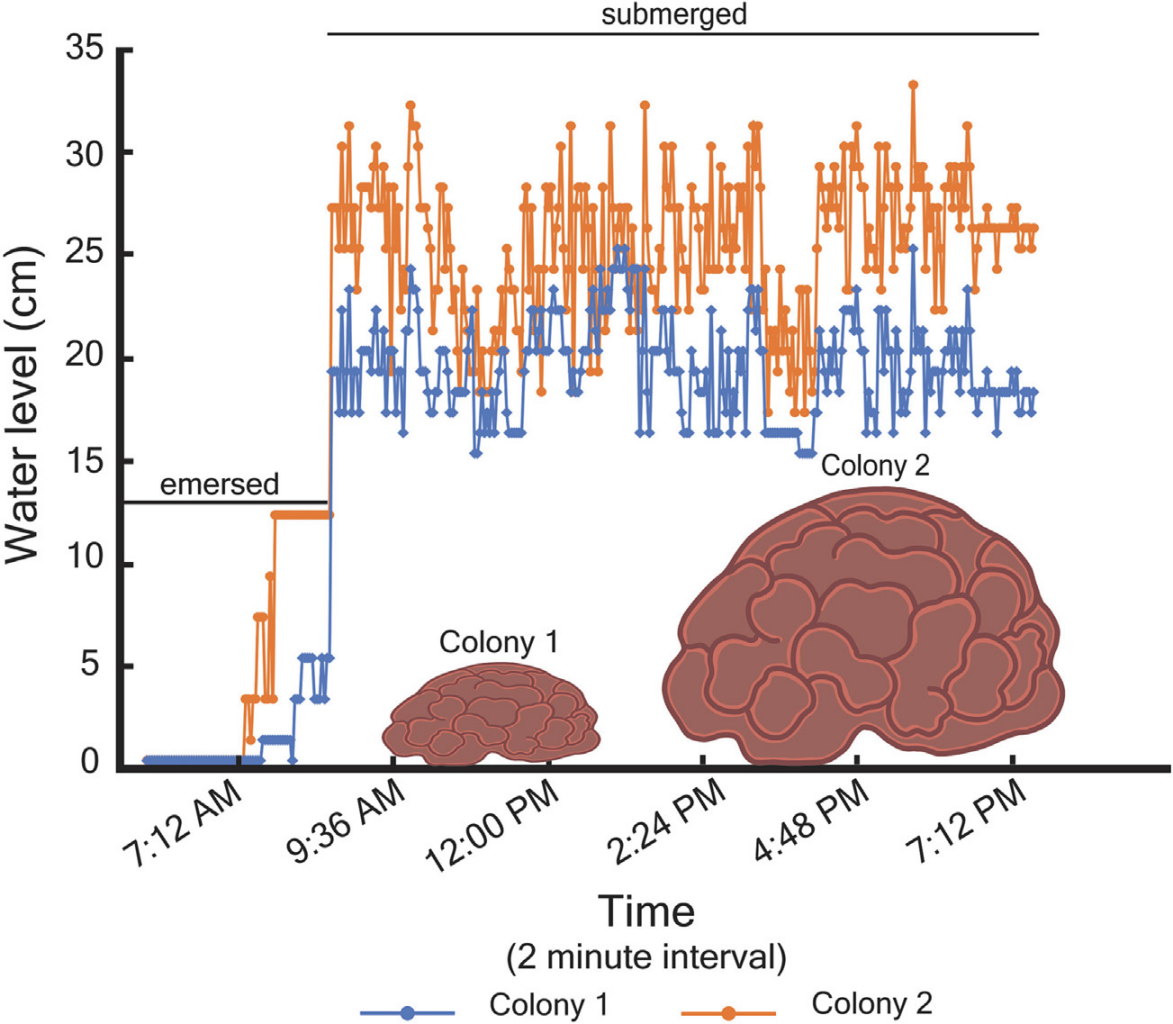
Results from field validation showing the instances when the coral colonies were emersed and submerged within the period of monitoring, based from changes in water level (derived from calculations of the difference between the fixed distance of ToF sensor to substrate and distances recorded by the sensor every time it hits the water surface). The logged data shows that the monitored coral colonies (i.e. Colony 1 and 2) are either ‘emersed’ or ‘submerged’ if, respectively, the calculated water level is lower or higher than the height of the colony [Colony 1 = 5 cm; Colony 2 = 14 cm].

The instances of emersion recorded by EmerSense (Fig. 4) coincided approximately with the projected low tidal heights for Pulau Hantu, on April 29 [9]. The coral colonies were emersed during the period 0630 h to 0900 h, with Colony 1 being fully submerged ∼ 30 min before Colony 2. Both colonies were submerged following the spike of tidal height around 0900 h until 1900 h.

**Fig. 5:**
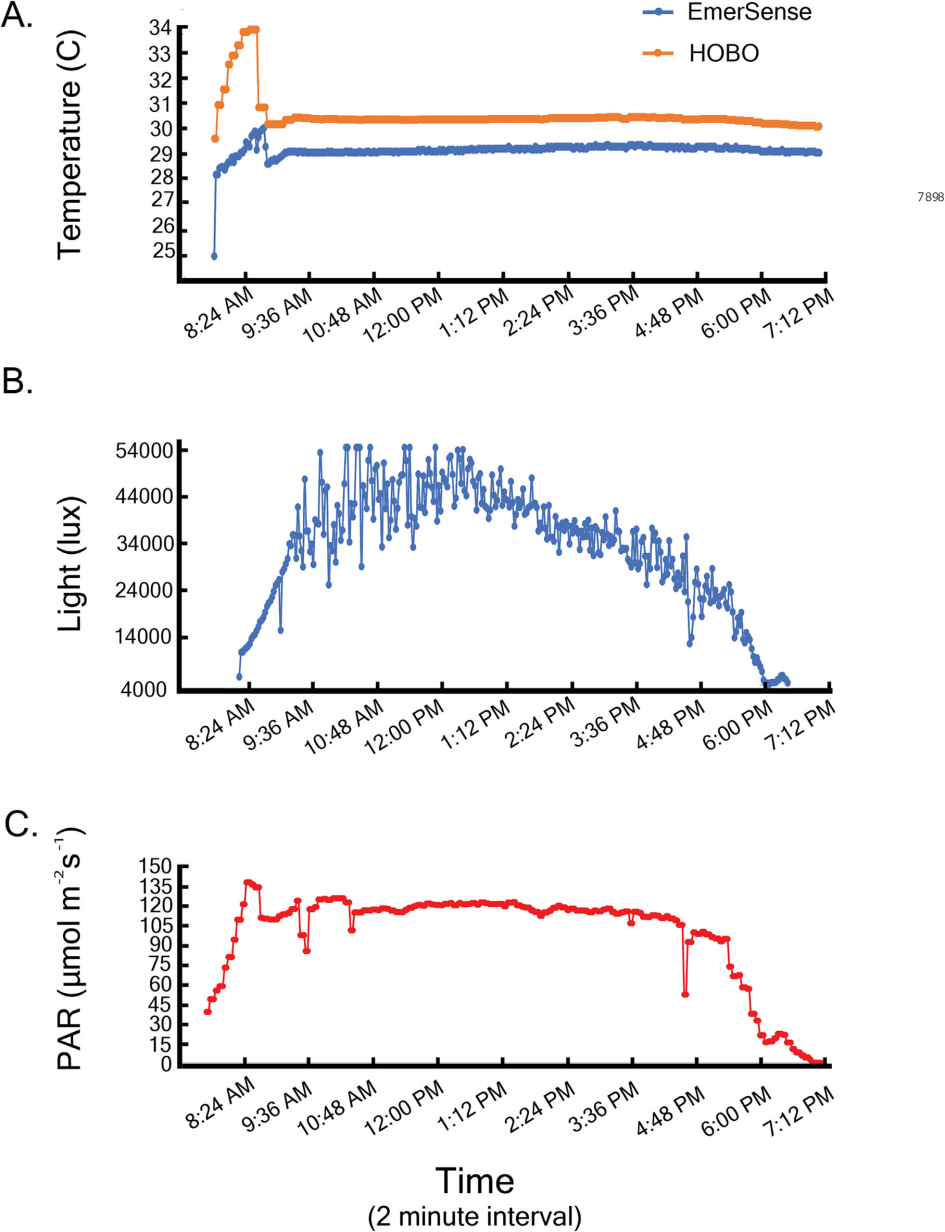
Environmental profile recorded by EmerSense (blue) in comparison with HOBO for temperature readings (A), EmerSense light readings in lux (B) and Odyssey light readings in PAR (C), recorded every two minutes. (For interpretation of the references to color in this figure legend, the reader is referred to the web version of this article.)

The results comparing ‘EmerSense versus HOBO temperature’ and ‘EmerSense versus Odyssey’ (Fig. 5A-B) showed similar patterns in terms of changes in temperature and light profile within the day. The temperature readings recorded by HOBO were, however, ∼1.3 °C (mean) higher than EmerSense.

## Discussion

The development of non-commercial low-cost environmental loggers for coastal applications has gained popularity in recent years [10], [11], [12], not only because of the availability and affordability of the required electronic components, especially microcontrollers, but also the degree of customizability that enables researchers to aquire data and implement their projects according to their research needs [13], [14], [15]. However, the corrosive nature of saltwater presents a challenge for monitoring environmental conditions in the marine environment as logging devices need to be waterproofed to protect internal electronics. This adds an additional financial investment that might make them inaccessible to research groups with constrained resources.

Here, we designed and tested a multiparameter logger, EmerSense, as a low-cost means of monitoring environmental dynamics in intertidal zones. Out of the three parameters it records, emersion is the most novel and important as these data are currently only available via tide-tables of variable accuracy (as they are based on predictions). Measuring the duration of emersion, and the time of day it occurs, is critical for understanding how intertidal sessile organisms respond to being exposed to air. In Singapore’s local context, this is particularly relevant to ongoing efforts to identify resilient corals for transplantation onto artificial coastal defences [5], [7].

The periods when the coral colonies were emersed coincided approximately with the projected low tide periods on 29 April 2021[9]. However, one of the unique features of EmerSense is the ability to determine all instances of emersion (i.e. partial or full emersion) at a particular location and time, by incorporating the height of a coral colony as a fixed reference to aid in calculations for post-processing of data. While Colony 1 and 2 were emersed around the same period i.e. 0630 h to 0900 h, the level of emersion was different due to the differing height of the colonies (i.e. Colony 1 = 5 cm; Colony 2 = 14 cm). The larger colony (i.e. Colony 2) was still partially emersed at 0830 h while the Colony 1 was already fully submerged (see Fig. 4). It is important to note that, unless the sea is absolutely calm, there will be some error into the water level measurements related to sea-surface quality. This may become more of an issue if sea foam or large waves are present. To reduce such noise, we suggest setting the logging interval to more frequent sampling (e.g. every two seconds) and use running averages of readings over one to two minute periods.

Light and temperature profiles measured using EmerSense closely tracked those from the Odyssey (light) and HOBO (temperature) loggers respectively, although the absolute values were different (see Fig. 5A-C). This is likely due to differences in specifications of sensors and other materials used (e.g. housing) or a combination thereof. The light sensor used for EmerSense is more readily available and inexpensive but, unlike Odyssey, it measures light intensity in lux. Lux can be converted into PAR using a conversion factor, however, this can be inaccurate and should be performed with caution [16], [17]. Further, the Odyssey sensor accounts for differences in the angle of incoming light as well as scattering and attenuation [18]. Light loggers that measure in lux are still widely-used for marine research, including those on photosynthetic organisms [19], [20], [21].

Temperatures measured by EmerSense were generally lower compared to the HOBO logger, which may also have been due to differences in sensor and housing materials. EmerSense features a low-cost temperature sensor that is a silicon bandgap thermometer based on bipolar junction transistors and is primarily designed for industrial applications and more extreme environments [22]. Its accuracy largely depends on the range of temperature being measured ie. ± 0.5 °C within the range of -20 °C to 50 °C, increasing to ± 2 °C for the full sensor range of -55 °C to 125 °C [23], [24]. While this can be a slight disadvantage, temperature readings can be calibrated against precision thermal sensors such as a high-quality thermistor [25]. Additionally, the accuracy of the device can be further improved through a calibration technique based on a curvature correction at multiple reference temperatures [24].

Overall, the multiparameter logger EmerSense we designed presents a low-cost alternative for *in situ* monitoring in aquatic systems. To our knowledge, it is the first low-cost emersion logger. The model described here includes light and temperature sensors that provide information about the surrounding conditions during emersion events. EmerSense could be further customized to include other sensors (e.g. pH, turbidity sensors) to measure and log environmental data to suit the needs of the research project but pre-deployment tests and calibration are highly recommended. Although they are more expensive, there are alternative temperature and light sensors that can be considered and incorporated into EmerSense, such as a Resistance Temperature Detector (e.g. PT1000 and MAX31865) and a RGBC light sensor (e.g. TCS34725), respectively. These are potentially more accurate than the components used here, but we have not tested them ourselves. The power source can be replaced with a rechargeable system if required. A global system for mobile communication (GSM) could also be installed to relay real-time data to the end user. We demonstrated how EmerSense may be applied to coral reef research in Singapore, however, we anticipate it will be useful for intertidal projects worldwide. It can aid the establishment of baselines for a range of resource management applications where tidal fluctuation is a key variable. As coastal systems exhibit wide temporal and spatial gradients, EmerSense can help with location-specific observations to identify suitable restoration sites. As many such projects are occurring in developing countries, the low-cost of EmerSense will help expand management capacity, as well as benefit research projects with limited budgets.

## Ethics statement

This work does not involved human subjects and experiments directly on animals.

Specifications table

**Table:** 

Hardware name	“EmerSense”
Subject area	Biological Sciences (Ecology)
Hardware type	Field Measurements and Sensors
Open Source License	Creative Commons Attributions 4.0 International
Cost of Hardware	$70.77 (USD)
Source File Repository	https://doi.org/10.5281/zenodo.5158903

## CRediT authorship contribution statement

**Rosa Celia Poquita-Du:** Conceptualization, Methodology, Investigation, Formal analysis, Data curation, Writing – original draft, Writing – review & editing, Visualization. **Ian Peter Morgia Du:** Conceptualization, Methodology, Software, Investigation, Formal analysis, Data curation, Writing – review & editing, Visualization. **Peter A. Todd:** Conceptualization, Methodology, Writing – review & editing, Funding acquisition.

## Declaration of Competing Interest

The authors declare that they have no known competing financial interests or personal relationships that could have appeared to influence the work reported in this paper.
